# Salmagundi

**Published:** 1887-03

**Authors:** 


### SALMAGUNDI.

EDITED BY E. G. BETTY, D.D.S.

DR. KARTULIS, of Alexandria, Egpyt, claims that the giant Amœbæ are the specific cause of dysentery.

RICHARD DONOVAN, of Conshohocken, Pa., whose son recently lost several front teeth by being kicked in the face by a horse belonging to Martin Devaney, was awarded $25 damages. He sued for $10,000.—*Clipping.*

IT is found that timber which has floated in water for some time (raft timber) is no longer liable to dry rot. The water slowly dissolves out the albumen and salts, thus depriving the fungus of the nutriment needed for its development.—*English Mechanic.*

M. GALLIPPE has related to the Paris Academy of Medicine the discovery of a new fungus composed of tubes and spores of mycelium developed in the human saliva. It belongs to the moniliæ family, and M. Gallippe has given it the name of *moniliœ sputicola.*—*The Microscope.*

THE publishers of the new monthly, *Western Dental Journal,* very wisely put friend Thompson in charge of the department devoted to "Journalistic Reviews and Selections," thereby saving confusion in the composing room by the printers endeavoring to decipher the hieroglyphics in which the distinguished editor is wont to express himself.

WE recently had occasion to remove a filling from the posterior proximal surface of a lower second molar, introduced a little over five years ago. The lower two-thirds of the filling was made with Robinson's textile foil, the balance with gold. Apparently the filling was all intensely black, but upon removal the blackening was found to be upon the exposed surface of the tin only.

Dr. David Christie, writing in the *Medical Press*, advocates the use of "iron alum" in saturated solution as a powerful styptic and antiseptic, causing wounds to heal in a very short time. He also uses it to prevent hemorrhage from small blood vessels during the course of a surgical operation. The "iron alum" may thus be valuable to the dentist in controlling obstinate alveolar hemorrhage after extraction.

---

A young blacksmith of Louisville, Ky., is reported to have invented or discovered a new method of hardening and tempering steel, by which hardness and elasticity are carried forward in combination. Many instances are given to show its superiority over steel made and tempered in the ordinary way. The invention is as yet a secret, not patented, but a company has been formed in that city for its manufacture. If this be true, then we can have drills and chisels that will cut very hard tooth substance without danger of breakage or dulling of the instrument, a desideratum of great value.

---

Malthe speaks highly of the following caustic and alterant treatment in torpid chronic ulcers and fistulæ. The surface in question is sprinkled with iodoform, then the lunar caustic is applied thoroughly, and finally the sprinkling with iodoform is repeated. A slight effervescence is produced with evolution of nitrous acid, iodine, iodide of silver, and perhaps also nitric acid and other combinations, all of which bodies act in their nascent state upon the tissues. The ulcers heal with comparative rapidity, and are protected by an antiseptic layer of iodoform and iodide of silver.—*Med. Chron.*

---

The first number of the *Western Dental Journal* was issued in January of this year. In color of cover, size of page and type, it is an almost exact counterpart of the *Review*, and, like it also, is supplied with associate editors. The variety of departments will make its contents interesting to the reader and of value to the dentist subscribing for a limited number of professional journals.

The recent issue of the two publications mentioned, is a matter of congratulation to the profession, inasmuch as it evidences the growth of dentistry in the West and Northwest, and shows that folks know something in those far off parts of the country, if they don't eat beans or ape the "blarsted Britisher."

---

A New Local Anæsthetic.— Drumine is the title of a new Australian local anæsthetic discovered and described by Dr. John Reid, of Port Germein, South Australia (*Lancet*, December 18, 1886). Euphorbia drummondii is the species from the milky juice of which the alkaloid drumine was prepared. Cocaine is known to have a mixed action on sensory and motor nerves, and causes preliminary excitement; whilst drumine is said to have an almost purely sensory paralyzing effect, and does not cause excitement. Experiments were made on cats and on the observer's tongue. It was injected into the legs of the former animals and caused general dullness, with marked impairment apparently of all forms of sensibility. Placed on the tongue, nostrils, and hand of the observer, the resulting anæsthesia was most marked. The alkaloid has no action on the pupil, and small doses given internally produce no constitutional effect. It has been employed successfully in subcutaneous injections for sciatica and sprains. The experimentation has so far been imperfect and incomplete. We hope soon to have a fuller account of this new alkaloid, and to be able to give further information thereon. The amount injected subcutaneously was four minims of a four per cent. solution. Dr. Reid anticipates a brilliant future for the drug in the domain of nervous and cerebral diseases.— *Medical and Surgical Reporter.*

---

Two new features appear this year in connection with the *Ohio State Journal of Dental Science.* First, the dropping of the word State from the name of the journal, possibly for two reasons, one of which is that popularly it has been confounded with a newspaper of the same name (excepting the *Dental Science* part) published at Columbus, O., and the other that, having reached its seventh volume, it does not like the implication that its circulation is confined largely to the State of Ohio. Second, the starting of a new department, yclept "What We See and Hear," edited by L. P. Bethel, D.D.S., a very commendable addition to other features of the journal.

It may be interesting in this connection to note the variety of captions, names, terms, or what you will, by which the miscellaneous department of the various journals is designated: "Hints and Queries," "Queries," "Miscellany," "Our Aftermath," "Salmagundi," "Excerpta," etc., etc. SALMAGUNDI, by the way, is the most original single word adopted by any, and shared the name with but one other publication (until a few months ago), viz., *The Manhattan Magazine*, a literary production exclusively. The name was first used by Captain Marryat, the great delineator of English naval life, and subsequently exalted to literary distinction by our own charming Washington Irving. It may also be stated that the writer was unaware of the application of the word to the miscellaneous department of any publication until the REGISTER had been using it for nearly three years. Its popularity with our readers and friends is amply evidenced by the abreviations, "Sally," "Sal," etc., just as Goldsmith is proved to have entered the hearts of his friends and worshipers by their always applying to him the affectionate epithet of "Poor Goldy;" at the same time, we are not so ambitious as to have "Sally" mentioned in the same breath with "Goldy," yet we are glad to be spoken of so kindly, and hope the future has many good things in store for all of us.

### Correction.

In the REGISTER for November, 1886, on page 537, in an article on Dental Education, by Dr. B. H. Catching, in the third line, "district schools" should read "distinct schools;' and on page 539, eleventh line from top, "chemical medicine" should read "clinical medicine."

